# Evaluation of an Integrated Smart Sensor System for Real-Time Characterization and Digitalization of Postoperative Abdominal Drain Output: A Pilot Study

**DOI:** 10.1177/15533506211031459

**Published:** 2021-11-16

**Authors:** Mario V. Roser, Alexander H. R. Frank, Lea Henrichs, Christian Heiliger, Dorian Andrade, Laura A. Ritz, Jan Sabo, Andreas Rauschmayr, Oliver Muensterer, Jens Werner, Wojciech Konrad Karcz, Michael F. Berger

**Affiliations:** 1 Department of Pediatric Surgery, Dr. von Hauner Children’s Hospital, 9183 Ludwig-Maximilians-University Munich, Germany; 2 Department of General, Visceral, and Transplantation Surgery, 9183 Hospital of the Ludwig-Maximilians-University Munich, Germany; 3 Division of Pediatric Surgery, Department of General, Abdominal and Transplant Surgery, Essen University Hospital, Germany

**Keywords:** surgical drains, abdominal surgery, postoperative care, surgical innovation, spectroscopy, digital healthcare, digital patient, real-time monitoring

## Abstract

*Background*: For centuries, surgeons have relied on surgical drains during postoperative care. Despite all advances in modern medicine and the area of digitalization, as of today, most if not all assessment of abdominal secretions excreted via surgical drains are carried out manually. We here introduce a novel integrated Smart Sensor System (*Smart Drain*) that allows for real-time characterization and digitalization of postoperative abdominal drain output at the patient’s bedside. *Methods*: A prototype of the *Smart Drain* was developed using a sophisticated spectrometer for assessment of drain output. The prototype measures 10 × 6 × 6 cm and therefore easily fits at the bedside. At the time of measurement with our *Smart Drain*, the drain output was additionally sent off to be analyzed in our routine laboratory for typical markers of interest in abdominal surgery such as bilirubin, lipase, amylase, triglycerides, urea, protein, and red blood cells. A total of 45 samples from 19 patients were included. *Results*: The measurements generated were found to correlate with conventional laboratory measurements for bilirubin (r = .658, *P* = .000), lipase (r = .490, *P* = .002), amylase (r = .571, *P* = .000), triglycerides (r = .803, *P* = .000), urea (r = .326, *P* = .033), protein (r = .387, *P* = .012), and red blood cells (r = .904, *P* = .000). *Conclusions*: To our best knowledge, for the first time we describe a device using a sophisticated spectrometer that allows for real-time characterization and digitalization of postoperative abdominal drain output at the patient’s bedside.

## Summary

We introduce an integrated Smart Sensor System (*Smart Drain*) which spectroscopic measurements correlated significantly with conventional laboratory measurements for bilirubin, lipase, amylase, triglycerides, urea, protein, and red blood cells. The importance of this medical device innovation is that it allows for real-time characterization of postoperative abdominal drain output at the patient’s bedside.

## Introduction

### Background

Many of today’s surgical disciplines rely on drains to postoperatively evacuate fluid from the wound cavity. The evacuated fluid is then collected within the drain bowl for exudate storage and subsequent manual evaluation by visual inspection or laboratory analysis within larger time intervals.^1^ In this way, quantity and quality of drainage output continue to be valuable proxies for monitoring the postoperative healing process following a large array of abdominal surgeries.^2^ However, as important as this information may be, the practice of manual processing of drain fluids has evolved little to none over the last decades and continues to be associated with significant problems, such as clogging,^3^ imprecise documentation,^4^ delayed removal and analysis of drain fluid,^5^ a high protocol workload for nursing staff^6^ as well as, most importantly, missed or delayed recognition of changes in quality of drain output indicating serious complications such as infection, bleeding, pancreatic fistulas,^7^ bile leaks, and intestinal perforations.^8^

### Aim

The aim of this study was to address and alleviate the clinical challenges of today’s drain management as stated above by developing a medical sensor device (*Smart Drain*) capable of transforming today’s manual drain monitoring toward a continuous real-time, digitalized monitoring all awhile assuring precision, efficacy, and—in the long run—cost-effectiveness.

## Material and Methods

### Prototype Development

Since the quantity of drain output can easily be measured using an integrated flowmeter or a scale, this study focuses primarily on the characterization of the quality of the drain output using a sophisticated yet affordable and compact spectrometer. The spectrometer used in the prototype offers fast and non-obstructive measurement in the detection range between 280 nm (near-ultraviolet) until 940 nm (near-infrared) with values being captured by 19 discrete detection channels. It thus exceeds the perception of the human observer at both ends of the visible spectrum (roughly 380 nm until 700 nm). The spectrometer was combined with an array containing 17 different exposure configurations emitting light at wavelengths matching the respective detection channels. The *Smart Drain* thus outputs a 17 × 19 = 323 data matrix containing exposure values influenced by the absorption of the drain fluid. This data matrix amounts to a spectrometric fingerprint of the fluid sample.

The prototype builds upon the existing workflow of drain insertion and management within the operating room.

**Figure 1.**
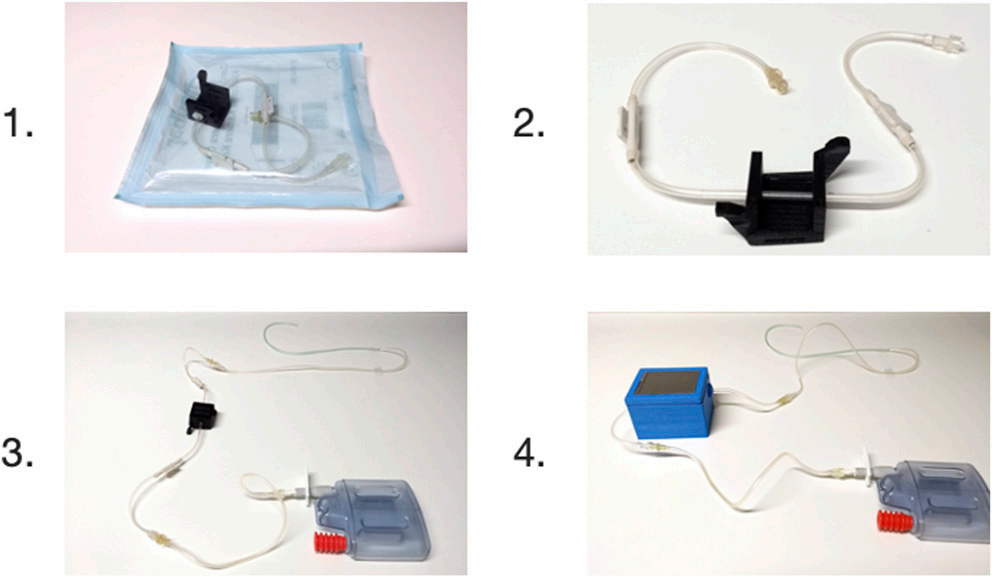
Handling and clinical application of the *Smart Drain*.

Once surgery is completed, a sterile, disposable tubing with the connector for the *Smart Drain* is handed to the surgeon in the operating room (see Figure 1—steps 1. and 2.). The surgeon merely needs to insert the tubing section in the drain line already being in place. One end is attached to the collecting container and the other end collects the fluid from the surgical site (see Figure 1—step 3.). To start data collection, the reusable analytics unit of the *Smart Drain* is magnetically attached to the connector (see Figure 1—step 4.).

### Study Population

A total of 45 drain fluid samples from 19 patients, 11 of them having multiple drains in place, were collected. 41 samples were collected from adult patients at the Department of General, Visceral, and Transplant Surgery while 4 samples came from pediatric patients at the Dr. von Hauner Children’s Hospital. Inclusion criteria were (1) informed consent, (2) recent abdominal surgery, and (3) one or multiple drains being in place. The study was approved by our ethics committee (project reference: 18-275) Table 1.

**Table 1.** Study Population.

Characteristic		Mean (Range)
Age	Years	53 (2–83)
Gender	Male/female	9/10
Diagnosis	Malignant/benign	13/6
	Carcinoma	7
	Sarcoma	2
	Neuroendocrine tumor	3
	Other tumor	2
	Other (e.g., ileus and hernia)	5
Operative intervention	Elective/emergence	18/1
	Primary	11
	Recurrence	4
	Revision	4
	Multivisceral resection	3
	Liver resection	3
	Pancreas resection	3
	Colon resection	2
	Tumor resection	2
	Other	6
Length of hospital stay	Days	24 (12–41)
Number of drains	Per patient	2 (1–4)
Location of drains	Abdominal	40

### Measurements

**Figure 2.**
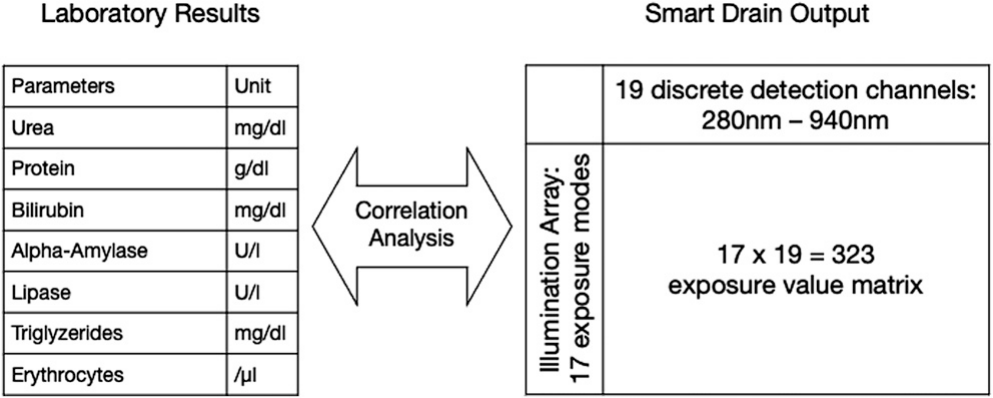
Correlation of validated laboratory results with exposure values.

Even though our prototype allows for direct integration into any standard drain tubing system (see Figure 1), in this pilot study, we carried out all measurements with drain fluid that was extracted from the drain container for this purpose. This was necessary in order to assure a control sample of the exact same composition as the sample analyzed by our *Smart Drain*. Therefore, while still being in place, the drains were tapped for a fluid sample and measured immediately. Where direct sample processing was not possible, samples were meanwhile stored at 4°C. In order to maintain a realistic scenario, no sample processing such as separation or filtration of any kind was applied. The samples were divided in two equal fractions. One thereof was sent to the clinical laboratory to obtain validated clinical reference parameters. The other fraction was sampled using the prototype of the *Smart Drain*. The exposure value matrix was subsequently analyzed for correlations with the obtained laboratory parameters using bivariate and partial correlation analysis (see Figure 2). Where the data quality seemed sufficient, the fit of several models to the data was evaluated using curve fitting. No data (outliers etc.) were intentionally excluded from the analysis. However, laboratory parameters published with “larger/smaller than (>/<)” can neither be processed with correlation analysis nor curve fitting. This occurred in 4 cases during the bilirubin analysis and in 7 cases during the erythrocyte analysis. Case numbers are also displayed in the correlation tables (see Supplementary Figures S1–S8). IBM SPSS v27 was used for all statistical analysis carried out. Plots were created using GraphPad Prism 9.

## Results

### Erythrocytes and Bilirubin

**Figure 3.**
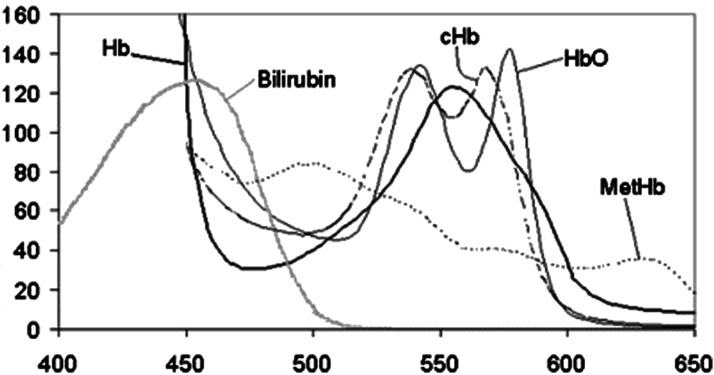
Absorption coefficients in mM^–1^cm^–1^ (Y-axis) for hemoglobin and bilirubin in the 400–650 nm range (X-Axis).^9^

We first focussed our attention on the analysis of detection of blood and bile as the primary indicators of interest for two reasons: First, the majority of our patient population underwent abdominal surgery where blood and bile are important postoperative indicators. Second, blood and bile are both substances that are known to have distinct optical characteristics making them suitable for spectroscopic detection.^9^ Hemoglobin present within the erythrocytes has absorption spectra varying depending on its carrier state (hemoglobin (Hb), oxyhemoglobin, carboxyhemoglobin, or methemoglobin). The absorption maxima are reported to occur between 525 nm and 575 nm as well as between 400 nm and 425 nm.^10^ Bilirubin has its absorption maximum at 450.5 nm (see Figure 3).

**Figure 4.**
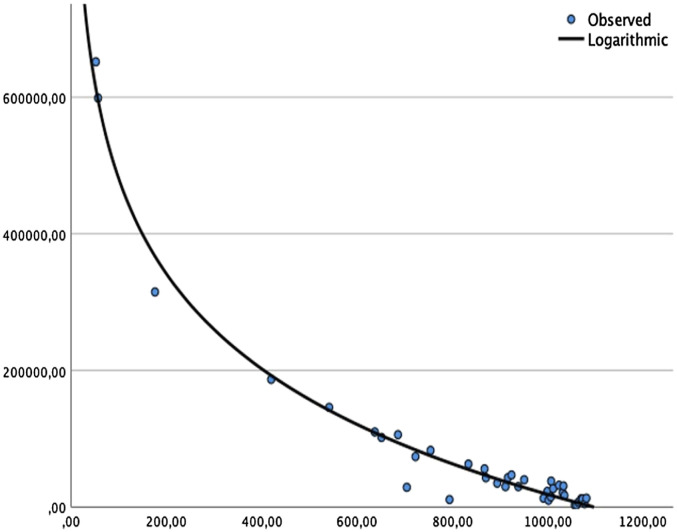
Logarithmic model predicting erythrocyte concentration (*Y*-axis) using spectrometer output at 560 nm (X-Axis).

To detect the erythrocyte concentration, we choose the 535 nm and 560 nm detector channels as they are closest to the described absorption maxima. We observed statistically highly significant correlation coefficients at both channels (see Supplementary Figure S1). The stronger correlation coefficient (−.904; Pearson’s r) occurred at the 560 nm channel. A curve fitting with several models (quadratic, cubic, and logarithmic) was conducted and produced several viable models, with the logarithmic model finally yielding the best fit (see Figure 4).

To detect the presence of bilirubin, we chose the 460 nm detector channel as it being closest to the absorption maximum. Subsequently, we observed a moderate negative correlation coefficient (r = −.507), which was highly significant (*P* = .001) between the bilirubin concentration as determined by the clinical laboratory and the *Smart Drain* spectrometer output at the 460 nm channel.

When looking at the overlapping spectra of erythrocytes and bilirubin in Figure 3, mutual conflicting measurements seem likely. Indeed, the correlation table for both substances and its associated detector channels show that erythrocytes concentration is not only registered on the respective detector channels but also rather strongly on the 460 nm bilirubin detector channel (see Supplementary Figure S2). However, in turn bilirubin concentration is sensed by the 560 nm detector channel.

This indicates that the presence of erythrocytes in the drain fluid could interfere with the bilirubin measurement. Therefore, to improve our understanding of this effect, we conducted a partial correlation while specifying the laboratory erythrocyte concentration as a control variable. In this manner, we analyzed all samples and observed a rise of the correlation coefficient to −.666 (see Supplementary Figure S3).

Since the erythrocyte concentration from the laboratory as a control variable would not be known to the *Smart Drain* during independent operation when predicting the presence of bilirubin, we used the 560 nm and 535 nm detector channels associated with the erythrocyte’s concentration as proxy control variables. The partial correlation produced a correlation coefficient of −.658 which was highly statistically significant *P* = 0.000 (see Supplementary Figure S4). It is noteworthy to point out that the use of the *Smart Drain’s* internal data for controlling the bilirubin measurement reproduced 98.8% of the correlation coefficient as compared to controlling with the laboratory result.

### Additional Findings

**Figure 5.**
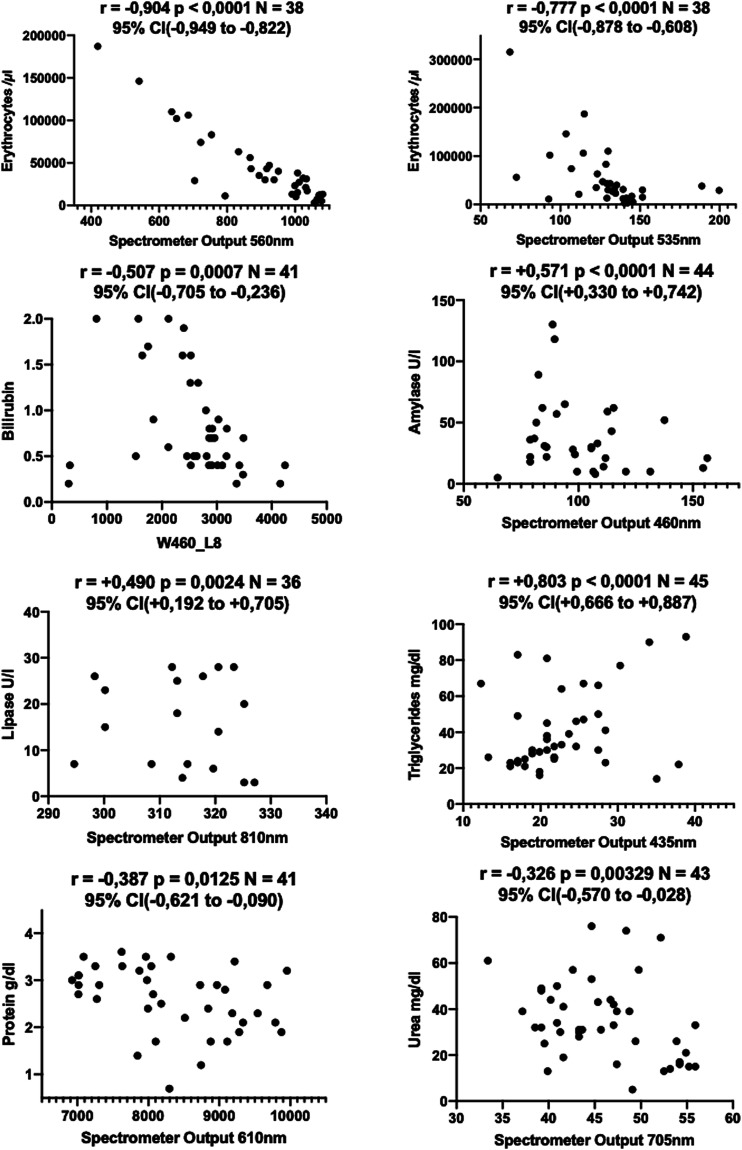
Data plots (scaled to fit for better visualization).

While correlating each of the 17 × 19 = 323 fields of the data matrix of the *Smart Drain* with the obtained laboratory parameters, we initially observed highly significant bivariate correlations for amylase, lipase, triglycerides, urea, and protein (Figure 5, plots 4–8).

The correlation for amylase (r = +.571, *P* = .001) appeared at 460 nm channel, which is also used for the bilirubin measurement. It is interesting to point out that, unexpectedly, the measurements do not conflict with each other (see Supplementary Figure S5). Most likely this can be attributed to the unique design of the *Smart Drain* where bilirubin and amylase are measured with different methods. Knowing from our previous analysis that the 460 nm channel potentially might be influenced by erythrocytes and bilirubin as well, a partial correlation was conducted specifying the respective internal control variables. We observed an increase of the correlation coefficient to +.650 which was highly significant (*P* = .000) (see Supplementary Figure S6).

The correlation for lipase appeared in the near-infrared spectrum at 810 nm (r = +.490, *P* = .002%). However, laboratory parameters for amylase and lipase nearly correlate perfectly (r = +.97). This does not come as a surprise considering their molecular makeup and the fact that both enzymes simultaneously indicate inflammation or injury of the pancreas. Subsequently, we observe that the lipase concentration does correlate with the 460 nm channel used to detect amylase. Vice versa, the amylase concentration correlates with 810 nm channel used to detect lipase (see Supplementary Figure S7). Based on these data, amylase and lipase activity could not be reliably separated.

Furthermore, bivariate correlations for triglycerides at 435 nm (r = +.803, *P* = .000), urea at 705 nm (r = −.326, *P* = .033), and protein at 610 nm (r = −.387, *P* = .012) were observed (see Supplementary Figures S8 and S9).

## Discussion

Despite all advances in the era of digitalization, to this day most if not all fluid samples collected in surgical drains around the world are being analyzed in an analogous fashion. This type of documentation is notoriously inaccurate, incomplete, labor-intense, and time consuming. Further, by design, results of charting or laboratory analysis are much delayed. We here demonstrate a functional prototype of an intelligent drain that can analyze fluid output from surgical drains using a spectrometer that delivers results continuously, in real-time and in a digitalized format. By analyzing 45 fluid samples from 19 patients, we demonstrated near perfect correlation coefficients for bilirubin, Hb, alpha-amylase/lipase, urea, protein, and triglycerides. The strength of the correlation coefficient in cases of bilirubin or alpha-amylase/lipase allows for a qualitative prediction and can safely state whether these compounds are present in the drain output or not. The strength of the correlation coefficient in case of the erythrocytes is most likely to allow for a quantitative prediction. This is underscored by the logarithmic modeling with an adjusted R-squared of 98.2%.

The clinical implications of the technique here are obvious. A recent study by Lyons et al. in 2015 revealed significant shortcomings in drain documentation. For example, their analysis showed that the 24 h flowcharting was missing in 35% of cases analyzed and a clear indication of the drain’s location at the patient’s body in 45%. Importantly, the recording of the type of the fluid output was missing in a 100% of the observed cases. Furthermore, events like flushing a clogged drain were not documented properly, which leads to misleading readings if the washing fluid is mistaken for regular drain output. While these numbers certainly cannot be generalized to other hospitals, it is obvious that documentation of drain output in an analogous fashion is a setup for inaccuracy and inconsistency. It is easily perceivable that the inability to appropriately monitor the output of surgical drains can lead to serious harm of the patient. This is especially true if changes in drain output quality are missed or delayed but would otherwise indicate an early pick up of a postoperative bleed or another serious complication.^8^ Also, improper documentation of surgical drain output may delay the removal of the drain and therefore the patient’s discharge from the hospital.^5^ The latter point is important to consider because it indicates how a type of intelligent drain that analyzes drain output digitally may improve the postoperative care not only of the few patients at risk for complications, but the much larger group of patients whose postoperative course will be uneventful. For this patient population, reassuring drain output analysis could steer medical decision-making toward early drainage removal and its associated benefits such as reduced infection risk and early discharge.^7^

One of the biggest advantages of an intelligent drain as we describe here is the provision of real-time, continuous and digital data. This type of analysis allows for easy integration of results into existing medical records of the patients and can be used for charting, documentation, billing, capacity planning, and in order to create “red flags” or another type of alerts provided in a digital fashion in order to indicate trouble. The *Smart Drain* with its 17 × 19 = 323 digital fluid fingerprint and with Wi-Fi, Bluetooth and 4G/5G connectivity already built-in in the current prototype is a capable and promising platform for cloud connection and cloud data processing. In this manner, we have integrated into our prototype the ability to have the results directly transmitted to any information system including any ordinary smartphone or smartwatch.

Further, in a larger vision, digitalized assessment and documentation of surgical drain output should be one additional data point incorporated into what is becoming the digitalized patient, along with vital signs and other patient characteristics. In the near future and using large sets of data points, artificial intelligence–based deep learning tools will allow for the creation of algorithms that can predict specific risk profiles of individual patients. An intelligent drain system as the one presented here takes into account these revolutionary developments. In our case, acquiring larger amounts of data enables the application of machine learning algorithms.

There are several limitations to our study. Importantly, the optical characteristics of some of the substances analyzed here are not yet described in the literature. We observed a strikingly high correlation for most substances, which is enormously encouraging. However, given the shortage of literature describing the optical characteristics of some substances in a setting as we describe it here, we cannot fully attain with certainty why these correlations occurred in the way they did. Furthermore, the limited number of samples used in this study cautions to make generalizations regarding the drain output in surgical fields other than abdominal surgery. Especially considering that drain fluid with its many possible fractions and enzymatic interactions is from a biochemical standpoint, a highly complex compound fluid. Also, at this point in our research and despite the obvious advantages that an intelligent drain system would have for the patient and within a health provider system, we cannot claim to what extend this will positively influence outcome. Therefore, further research with larger sample sizes and a better biochemical understanding are needed to validate the observed effects in specific clinical scenarios.

Having said that, we have successfully shown that a prototype whose detector chip’s cost amount to a mere US$12 is capable of reliably detecting the presence of bilirubin and other important markers in abdominal surgery and predict the concentration of erythrocytes in an artificial yet realistic laboratory setup. Given that our results are reported in real time allow for continuous assessment in a digitalized format, this development has the potential to substantially improve patient outcome.

## Supplemental Material

sj-tiff-1-sri-10.1177_15533506211031459 – Supplemental Material for Evaluation of an Integrated Smart Sensor System for Real-Time Characterization and Digitalization of Postoperative Abdominal Drain Output: A Pilot Study Click here for additional data file. Supplemental Material, sj-tiff-1-sri-10.1177_15533506211031459 for Evaluation of an Integrated Smart Sensor System for Real-Time Characterization and Digitalization of Postoperative Abdominal Drain Output: A Pilot Study by Mario V. Roser, Alexander H. R. Frank, Lea Henrichs, Christian Heiliger, Dorian Andrade, Laura A Ritz, Jan Sabo, Andreas Rauschmayr, Oliver Muensterer, Jens Werner, Wojciech Konrad Karcz and Michael F. Berger in Surgical Innovation

sj-tiff-2-sri-10.1177_15533506211031459 – Supplemental Material for Evaluation of an Integrated Smart Sensor System for Real-Time Characterization and Digitalization of Postoperative Abdominal Drain Output: A Pilot Study Click here for additional data file. Supplemental Material, sj-tiff-2-sri-10.1177_15533506211031459 for Evaluation of an Integrated Smart Sensor System for Real-Time Characterization and Digitalization of Postoperative Abdominal Drain Output: A Pilot Study by Mario V. Roser, Alexander H. R. Frank, Lea Henrichs, Christian Heiliger, Dorian Andrade, Laura A Ritz, Jan Sabo, Andreas Rauschmayr, Oliver Muensterer, Jens Werner, Wojciech Konrad Karcz and Michael F. Berger in Surgical Innovation

sj-tiff-3-sri-10.1177_15533506211031459 – Supplemental Material for Evaluation of an Integrated Smart Sensor System for Real-Time Characterization and Digitalization of Postoperative Abdominal Drain Output: A Pilot Study Click here for additional data file. Supplemental Material, sj-tiff-3-sri-10.1177_15533506211031459 for Evaluation of an Integrated Smart Sensor System for Real-Time Characterization and Digitalization of Postoperative Abdominal Drain Output: A Pilot Study by Mario V. Roser, Alexander H. R. Frank, Lea Henrichs, Christian Heiliger, Dorian Andrade, Laura A Ritz, Jan Sabo, Andreas Rauschmayr, Oliver Muensterer, Jens Werner, Wojciech Konrad Karcz and Michael F. Berger in Surgical Innovation

sj-tiff-4-sri-10.1177_15533506211031459 – Supplemental Material for Evaluation of an Integrated Smart Sensor System for Real-Time Characterization and Digitalization of Postoperative Abdominal Drain Output: A Pilot Study Click here for additional data file. Supplemental Material, sj-tiff-4-sri-10.1177_15533506211031459 for Evaluation of an Integrated Smart Sensor System for Real-Time Characterization and Digitalization of Postoperative Abdominal Drain Output: A Pilot Study by Mario V. Roser, Alexander H. R. Frank, Lea Henrichs, Christian Heiliger, Dorian Andrade, Laura A Ritz, Jan Sabo, Andreas Rauschmayr, Oliver Muensterer, Jens Werner, Wojciech Konrad Karcz and Michael F. Berger in Surgical Innovation

sj-tiff-5-sri-10.1177_15533506211031459 – Supplemental Material for Evaluation of an Integrated Smart Sensor System for Real-Time Characterization and Digitalization of Postoperative Abdominal Drain Output: A Pilot Study Click here for additional data file. Supplemental Material, sj-tiff-5-sri-10.1177_15533506211031459 for Evaluation of an Integrated Smart Sensor System for Real-Time Characterization and Digitalization of Postoperative Abdominal Drain Output: A Pilot Study by Mario V. Roser, Alexander H. R. Frank, Lea Henrichs, Christian Heiliger, Dorian Andrade, Laura A Ritz, Jan Sabo, Andreas Rauschmayr, Oliver Muensterer, Jens Werner, Wojciech Konrad Karcz and Michael F. Berger in Surgical Innovation

sj-tiff-6-sri-10.1177_15533506211031459 – Supplemental Material for Evaluation of an Integrated Smart Sensor System for Real-Time Characterization and Digitalization of Postoperative Abdominal Drain Output: A Pilot Study Click here for additional data file. Supplemental Material, sj-tiff-6-sri-10.1177_15533506211031459 for Evaluation of an Integrated Smart Sensor System for Real-Time Characterization and Digitalization of Postoperative Abdominal Drain Output: A Pilot Study by Mario V. Roser, Alexander H. R. Frank, Lea Henrichs, Christian Heiliger, Dorian Andrade, Laura A Ritz, Jan Sabo, Andreas Rauschmayr, Oliver Muensterer, Jens Werner, Wojciech Konrad Karcz and Michael F. Berger in Surgical Innovation

sj-tiff-7-sri-10.1177_15533506211031459 – Supplemental Material for Evaluation of an Integrated Smart Sensor System for Real-Time Characterization and Digitalization of Postoperative Abdominal Drain Output: A Pilot Study Click here for additional data file. Supplemental Material, sj-tiff-7-sri-10.1177_15533506211031459 for Evaluation of an Integrated Smart Sensor System for Real-Time Characterization and Digitalization of Postoperative Abdominal Drain Output: A Pilot Study by Mario V. Roser, Alexander H. R. Frank, Lea Henrichs, Christian Heiliger, Dorian Andrade, Laura A Ritz, Jan Sabo, Andreas Rauschmayr, Oliver Muensterer, Jens Werner, Wojciech Konrad Karcz and Michael F. Berger in Surgical Innovation

sj-tiff-8-sri-10.1177_15533506211031459 – Supplemental Material for Evaluation of an Integrated Smart Sensor System for Real-Time Characterization and Digitalization of Postoperative Abdominal Drain Output: A Pilot Study Click here for additional data file. Supplemental Material, sj-tiff-8-sri-10.1177_15533506211031459 for Evaluation of an Integrated Smart Sensor System for Real-Time Characterization and Digitalization of Postoperative Abdominal Drain Output: A Pilot Study by Mario V. Roser, Alexander H. R. Frank, Lea Henrichs, Christian Heiliger, Dorian Andrade, Laura A Ritz, Jan Sabo, Andreas Rauschmayr, Oliver Muensterer, Jens Werner, Wojciech Konrad Karcz and Michael F. Berger in Surgical Innovation

sj-tiff-9-sri-10.1177_15533506211031459 – Supplemental Material for Evaluation of an Integrated Smart Sensor System for Real-Time Characterization and Digitalization of Postoperative Abdominal Drain Output: A Pilot Study Click here for additional data file. Supplemental Material, sj-tiff-9-sri-10.1177_15533506211031459 for Evaluation of an Integrated Smart Sensor System for Real-Time Characterization and Digitalization of Postoperative Abdominal Drain Output: A Pilot Study by Mario V. Roser, Alexander H. R. Frank, Lea Henrichs, Christian Heiliger, Dorian Andrade, Laura A Ritz, Jan Sabo, Andreas Rauschmayr, Oliver Muensterer, Jens Werner, Wojciech Konrad Karcz and Michael F. Berger in Surgical Innovation
