# Supplementary figures and images for: Evaluation of an Integrated Smart Sensor System for Real-Time Characterization and Digitalization of Postoperative Abdominal Drain Output: A Pilot Study
Source: Surg Innov. 2021 Nov 16;29(3):438–45. doi: 10.1177/15533506211031459 (PMC9227954; doi:10.1177/15533506211031459)

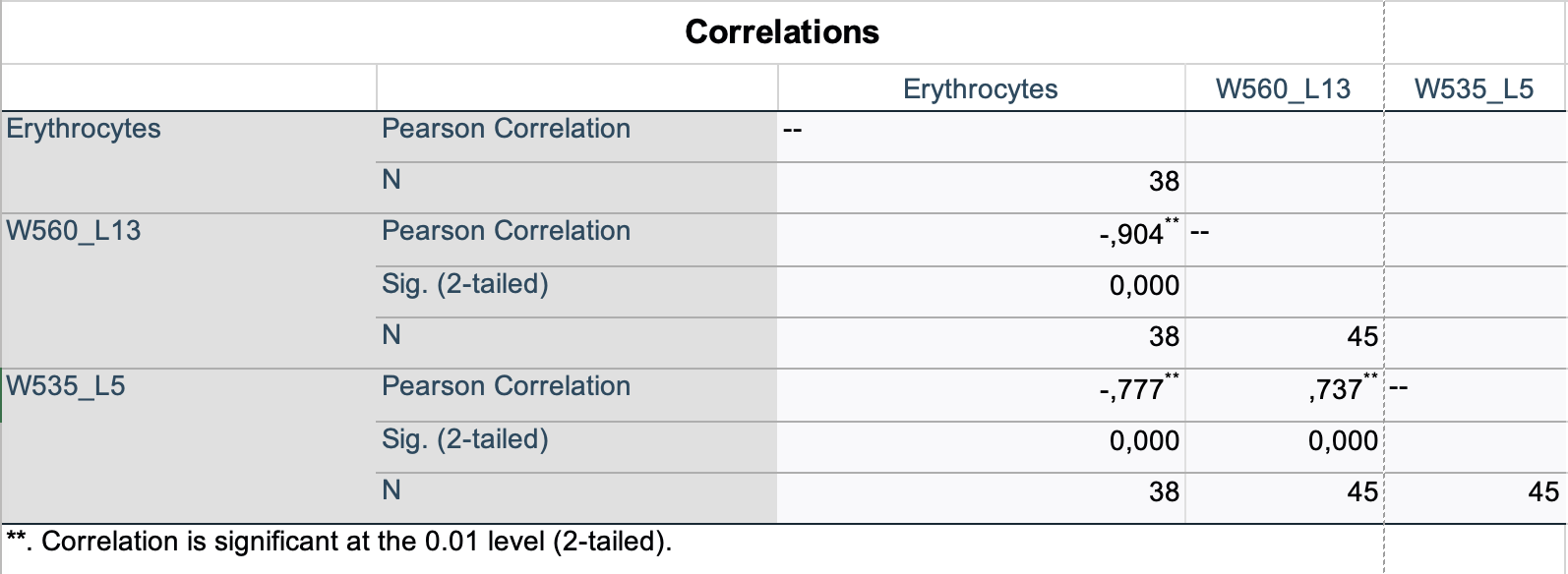

Supplement: sj-tiff-1-sri-10.1177_15533506211031459 – Supplemental Material for Evaluation of an Integrated Smart Sensor System for Real-Time Characterization and Digitalization of Postoperative Abdominal Drain Output: A Pilot Study [file sj-tiff-1-sri-10.1177_15533506211031459.tiff]

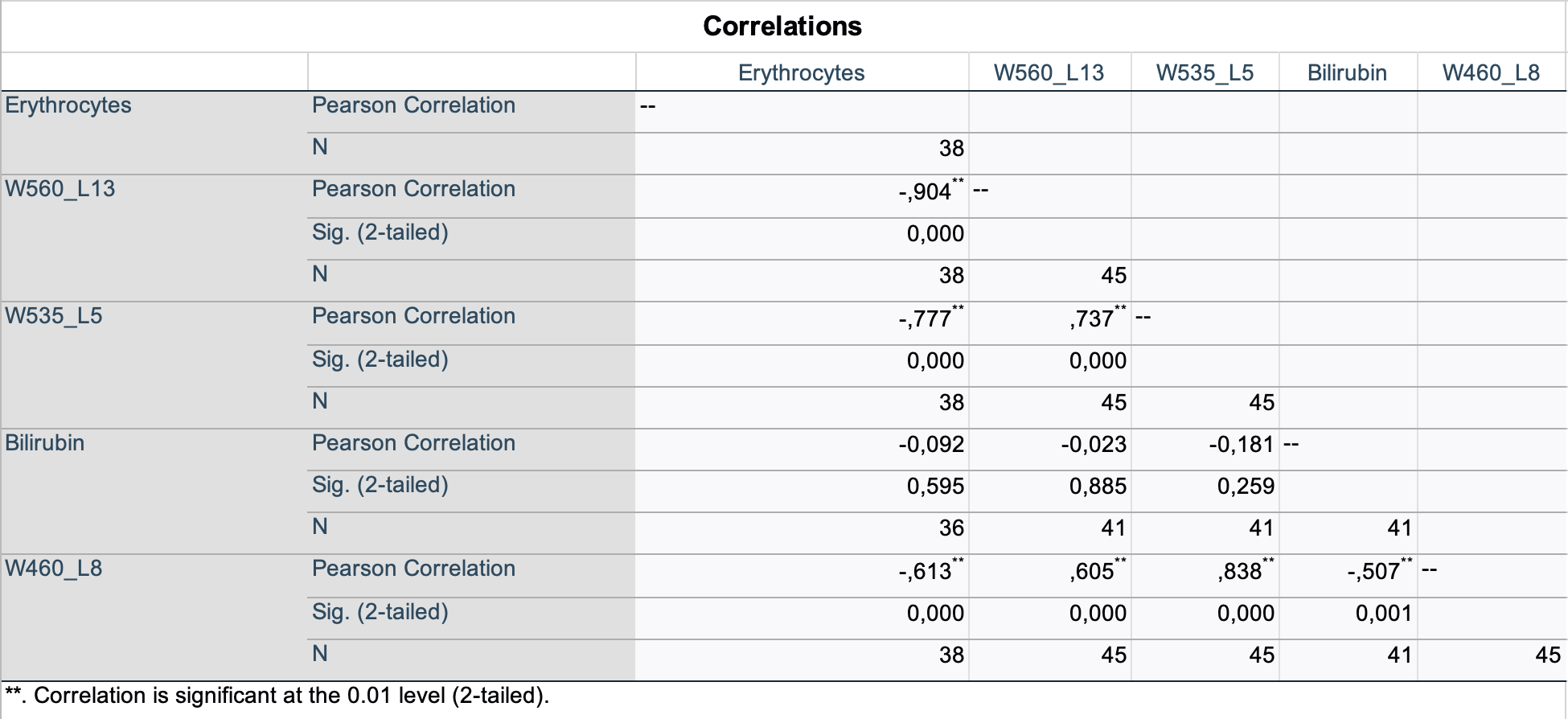

Supplement: sj-tiff-2-sri-10.1177_15533506211031459 – Supplemental Material for Evaluation of an Integrated Smart Sensor System for Real-Time Characterization and Digitalization of Postoperative Abdominal Drain Output: A Pilot Study [file sj-tiff-2-sri-10.1177_15533506211031459.tiff]

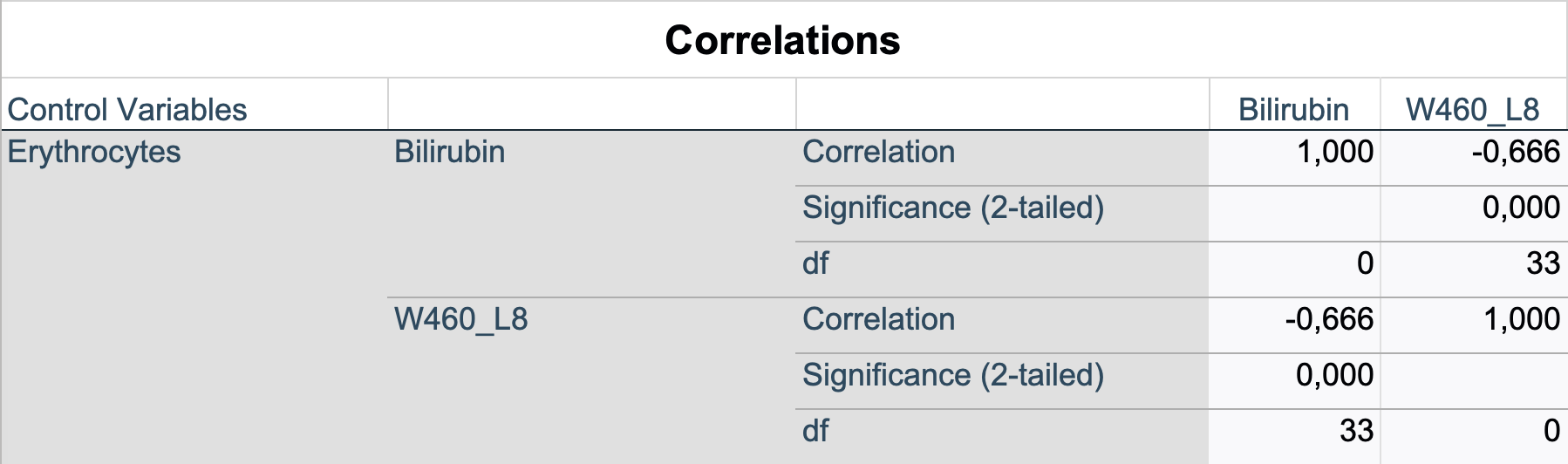

Supplement: sj-tiff-3-sri-10.1177_15533506211031459 – Supplemental Material for Evaluation of an Integrated Smart Sensor System for Real-Time Characterization and Digitalization of Postoperative Abdominal Drain Output: A Pilot Study [file sj-tiff-3-sri-10.1177_15533506211031459.tiff]

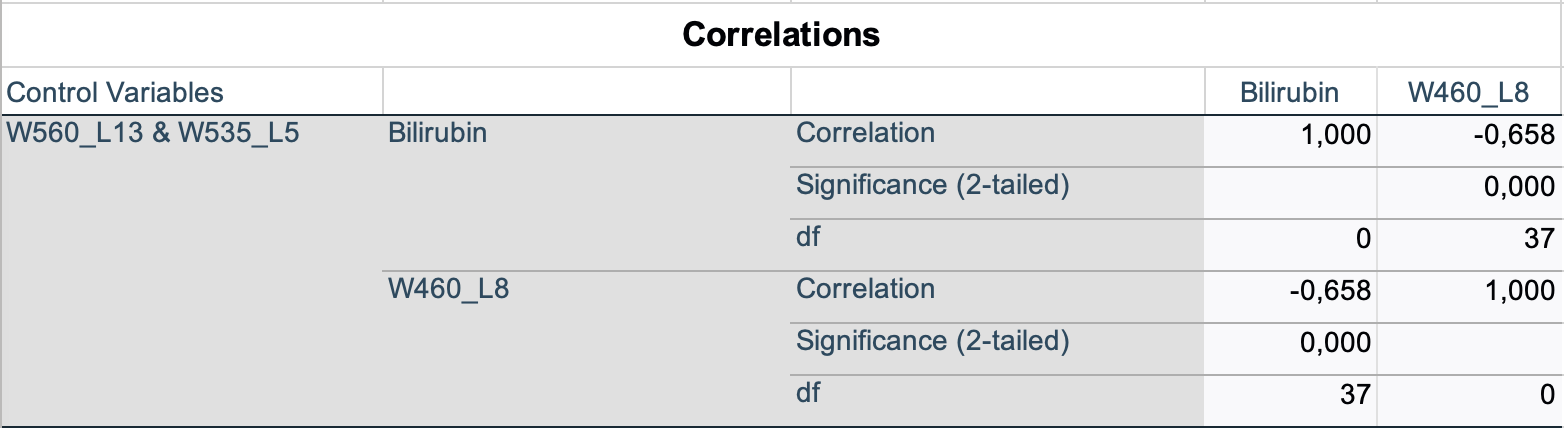

Supplement: sj-tiff-4-sri-10.1177_15533506211031459 – Supplemental Material for Evaluation of an Integrated Smart Sensor System for Real-Time Characterization and Digitalization of Postoperative Abdominal Drain Output: A Pilot Study [file sj-tiff-4-sri-10.1177_15533506211031459.tiff]

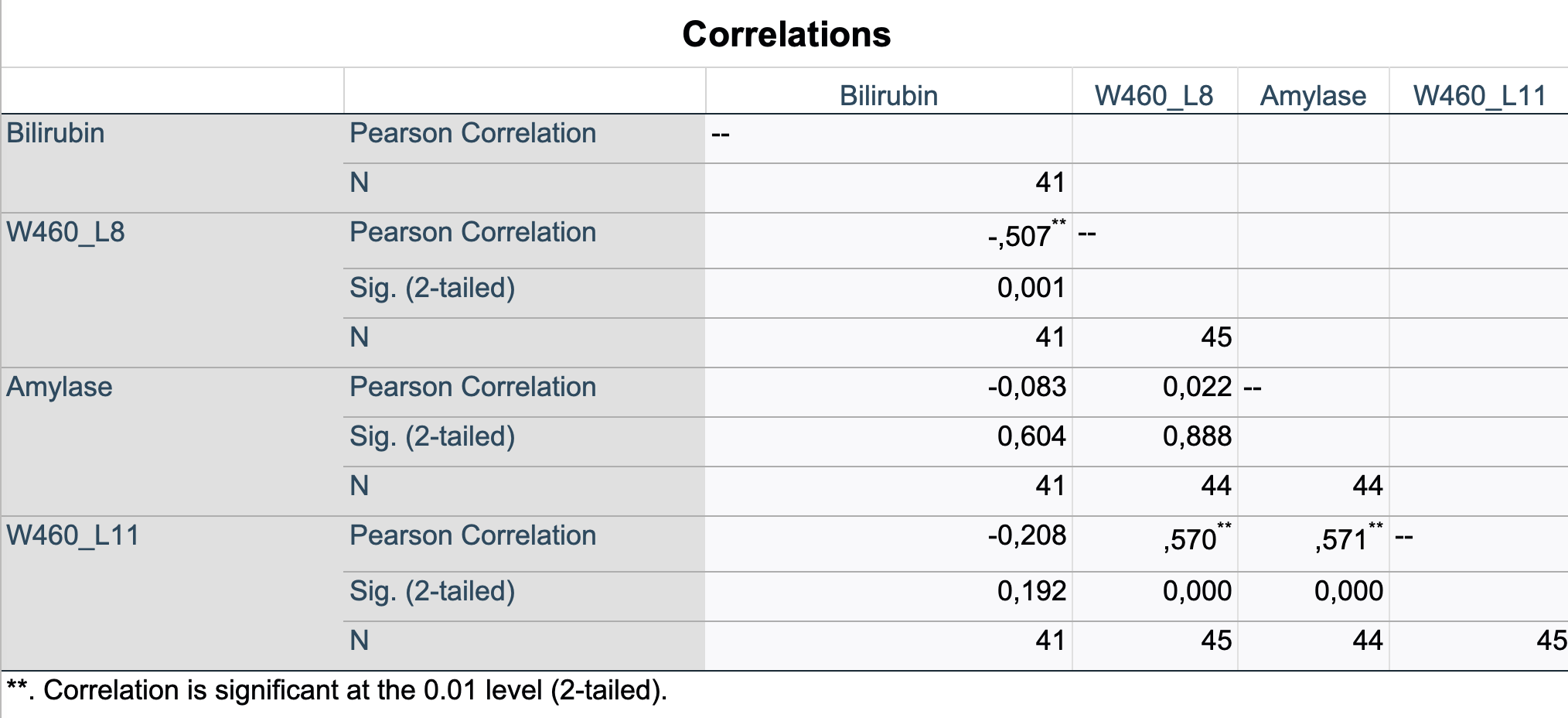

Supplement: sj-tiff-5-sri-10.1177_15533506211031459 – Supplemental Material for Evaluation of an Integrated Smart Sensor System for Real-Time Characterization and Digitalization of Postoperative Abdominal Drain Output: A Pilot Study [file sj-tiff-5-sri-10.1177_15533506211031459.tiff]

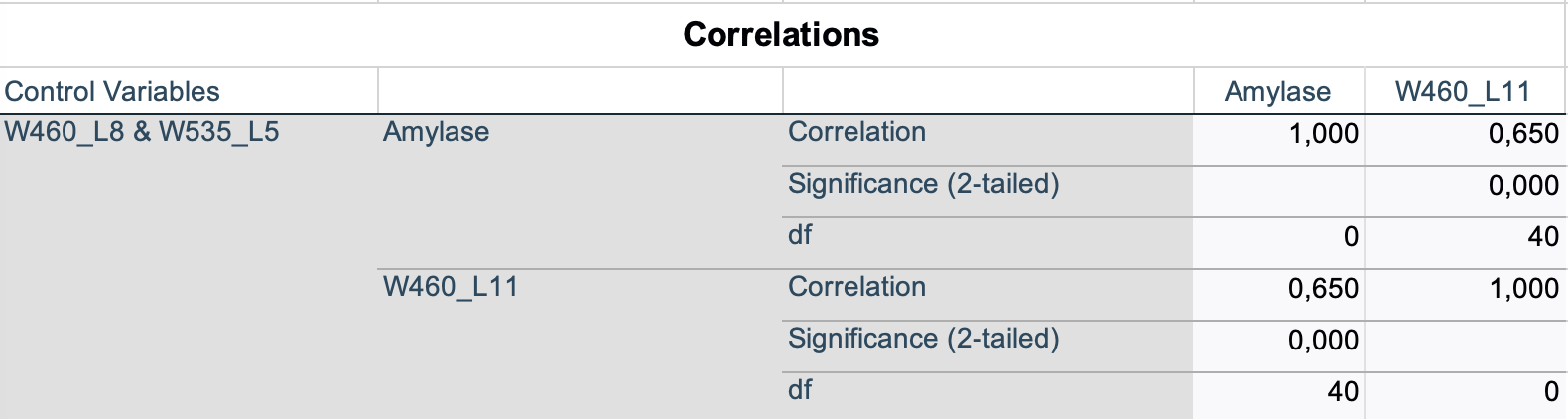

Supplement: sj-tiff-6-sri-10.1177_15533506211031459 – Supplemental Material for Evaluation of an Integrated Smart Sensor System for Real-Time Characterization and Digitalization of Postoperative Abdominal Drain Output: A Pilot Study [file sj-tiff-6-sri-10.1177_15533506211031459.tiff]

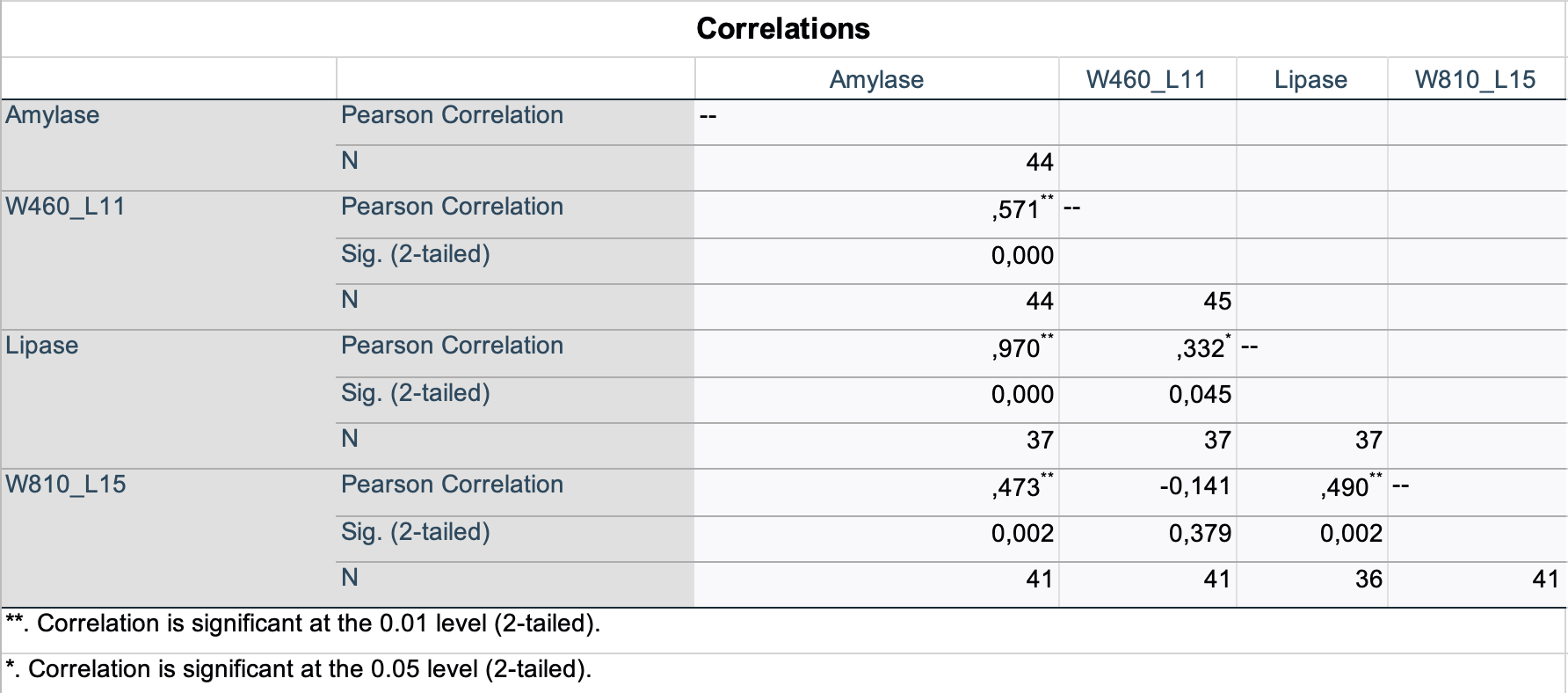

Supplement: sj-tiff-7-sri-10.1177_15533506211031459 – Supplemental Material for Evaluation of an Integrated Smart Sensor System for Real-Time Characterization and Digitalization of Postoperative Abdominal Drain Output: A Pilot Study [file sj-tiff-7-sri-10.1177_15533506211031459.tiff]

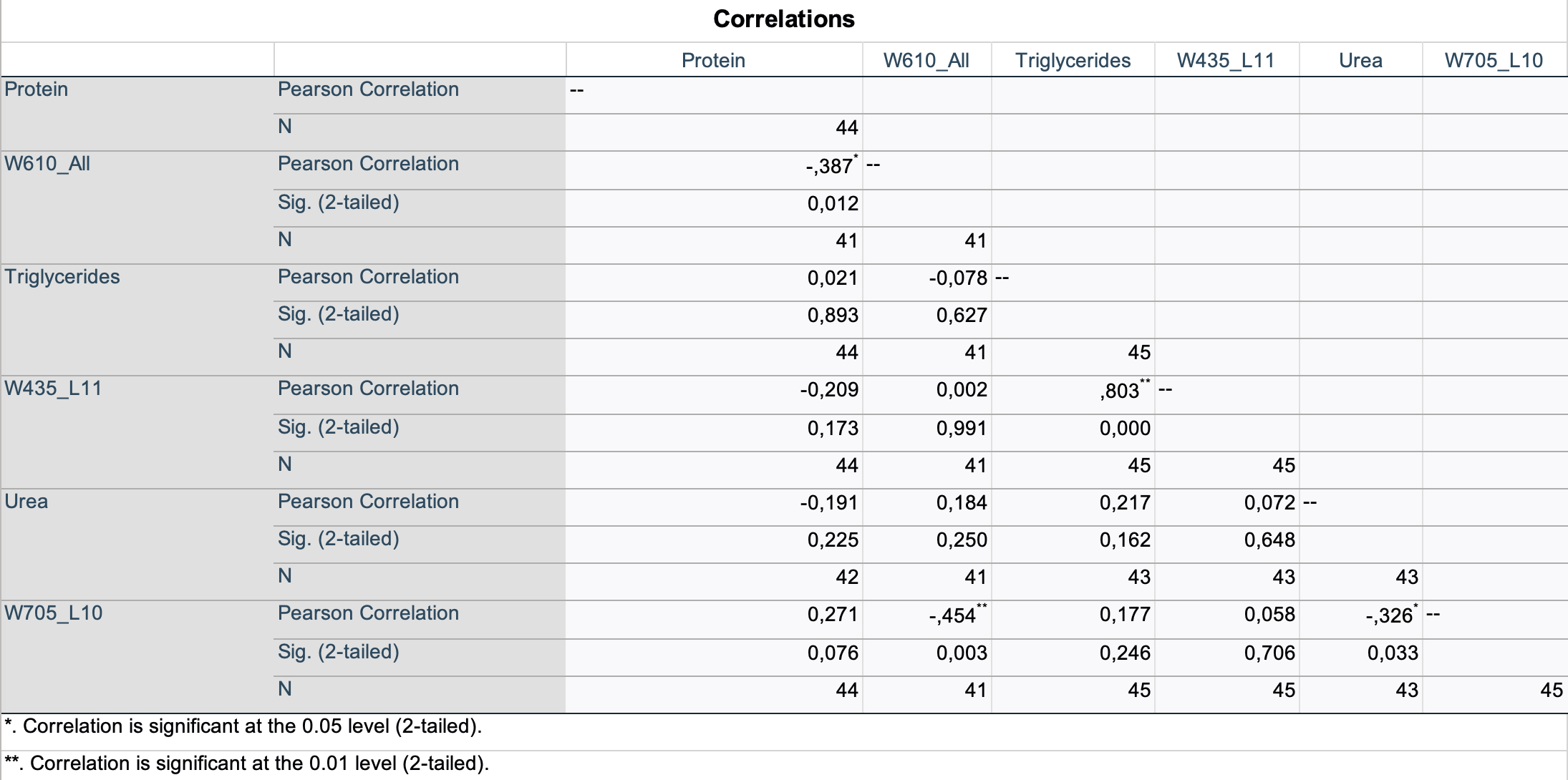

Supplement: sj-tiff-8-sri-10.1177_15533506211031459 – Supplemental Material for Evaluation of an Integrated Smart Sensor System for Real-Time Characterization and Digitalization of Postoperative Abdominal Drain Output: A Pilot Study [file sj-tiff-8-sri-10.1177_15533506211031459.tiff]

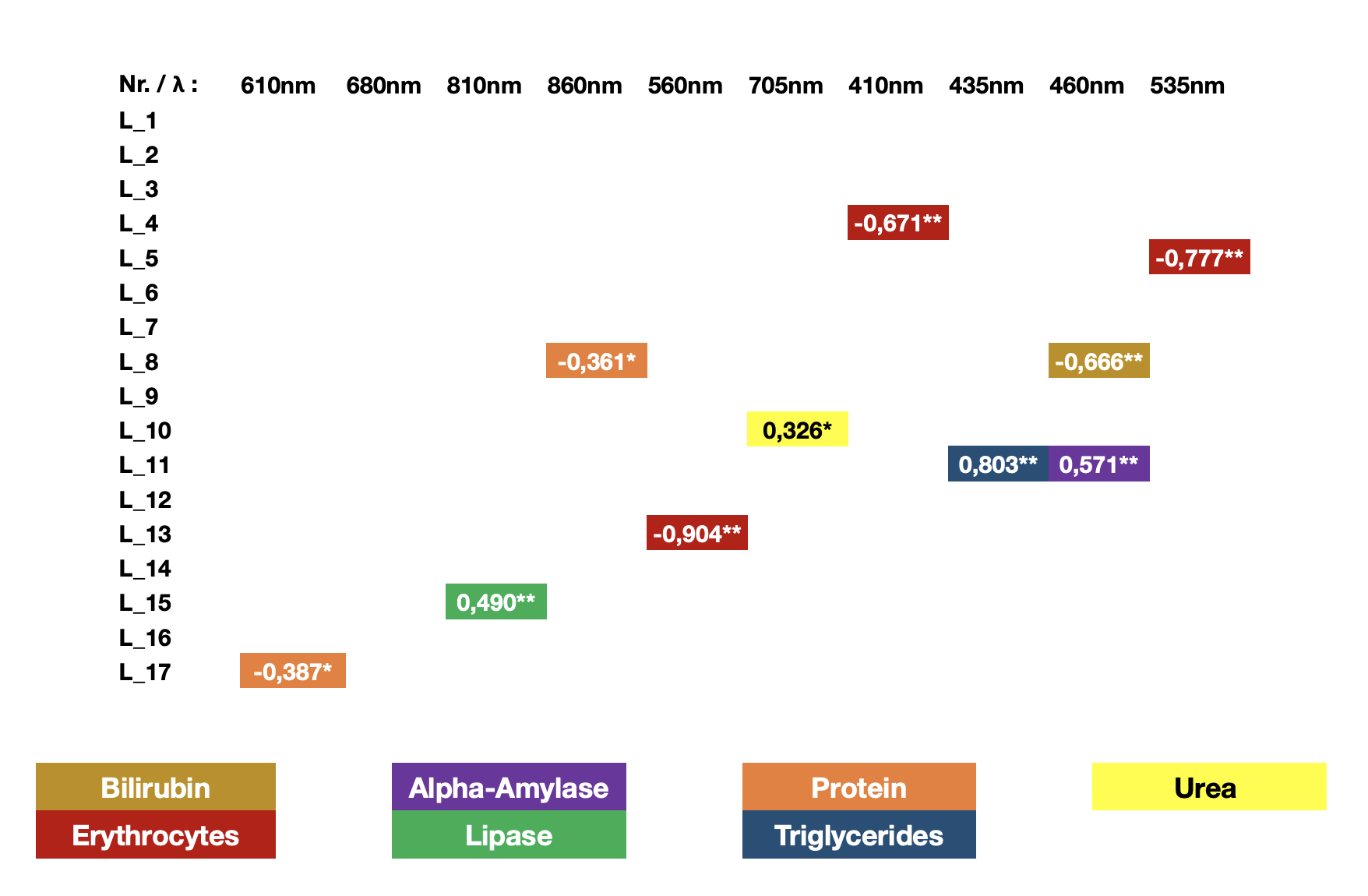

Supplement: sj-tiff-9-sri-10.1177_15533506211031459 – Supplemental Material for Evaluation of an Integrated Smart Sensor System for Real-Time Characterization and Digitalization of Postoperative Abdominal Drain Output: A Pilot Study [file sj-tiff-9-sri-10.1177_15533506211031459.tiff]
